# Ol. Morrhuæ

**Published:** 1853-03

**Authors:** 


*Ol. Morrhuæ.*—In Sweden, cod-liver oil has for some time been employed as an external application in the treatment of cutaneous maladies, and from that country it has recently been introduced into English practice. We have had frequent opportunities of watching its effects as prescribed by Mr. Paget, in the out-patient's room of St. Bartholomew's Hospital. The class of cases for which it appears most applicable, is that of chronic eczematous eruptions, unattended by acute inflammation of the skin or general pyrexia. In abating the troublesome itching which frequently accompanies this disease, especially in old people, it has manifested powers decidedly superior to those of any other application with which we are acquainted. In the majority of instances it can, of course, only be expected to assist constitutional treatment, not to supersede it.

We have as yet had no opportunity of forming an opinion respecting the proposed inunction of the oil in cases of phthisis, where the stomach cannot take it. Whilst on this subject, however, we feel inclined to strongly recommend the practice of exhibiting it *simultaneously with tonics*, as iron, quinine, etc., which is now adopted with great success at several hospitals. The digestion and assimilation of the oil appears to be much aided by such combination. In the treatment of cutaneous struma and lupus at the Hospital for Skin Diseases, it is usual to administer along with the oil small doses of mercurials, which are often continued for many months. The success attending this practice is very great, and appears to much exceed that which results from the administration of either drug alone.—*Lond. Med. Times.*

